# Comparative Analysis of General-Purpose vs. Domain-Specific Multimodal Models for Diabetic Retinopathy Classification

**DOI:** 10.3390/diagnostics16101504

**Published:** 2026-05-15

**Authors:** Mohammad Iqbal Nouyed, Mohammad Al-Mamun, Donald A. Adjeroh, Gangqing Hu

**Affiliations:** 1 Department of Microbiology, Immunology and Cell Biology, West Virginia University, Morgantown, WV 26506, USA; monouyed@hsc.wvu.edu; 2 School of Systems Science and Industrial Engineering, Binghamton University, State University of New York, Binghamton, NY 13902, USA; malmamun1@binghamton.edu; 3 Lane Department of Computer Science and Electrical Engineering, West Virginia University, Morgantown, WV 26506, USA; donald.adjeroh@mail.wvu.edu; 4 Department of Dermatology, West Virginia University, Morgantown, WV 26506, USA; 5 WVU Cancer Institute, West Virginia University, Morgantown, WV 26506, USA

**Keywords:** diabetic retinopathy, fundus images, image classification, large multimodal models

## Abstract

**Background/Objectives:** General-purpose and domain-specific multimodal foundation models show considerable promise in medical image analysis. In this study, we evaluated the classification accuracy of diabetic retinopathy vs. normal fundus images using general-purpose conversational models (Gemini 3 Flash, GPT-5.2, and Pixtral-Large), a medical conversational model (MedGemma-1.5), and its image-encoder (MedSigLIP), as well as ophthalmology-specific models (RETFound and EyeCLIP). **Methods:** We applied zero-/few-shot to general-purpose conversational models, linear probing, and fine-tuning approaches to domain-specific models for evaluation purposes. **Results:** We found that the zero-shot accuracies for Pixtral-Large (70.7%) and fine-tuned RETFound (77.1%) were comparable but lower than those of GPT-5.2 (77.9%), MedGemma-1.5 (88.2%), and Gemini 3 (88.5%) as well as the fine-tuned EyeCLIP (85.8%) and MedSigLIP (94.8%). The accuracy gains from few-shot prompting were substantial for Pixtral-Large (+7.4%) but were limited for GPT-5.2 (+3.6%), Gemini 3 (−3.4%), and MedGemma-1.5 (−1.1%). Embedding-based linear probing further improved accuracy over fine-tuning for RETFound (+9.7%) and yielded only marginal gains for EyeCLIP (+2.3%) but did not benefit MedSigLIP (−0.8%). Overall, with minimal prompting enhancement, general-purpose conversational models such as Gemini 3 and GPT-5.2 achieved performance comparable to ophthalmology-specific models that were either fine-tuned or enhanced via embedding-based linear probing, but remained inferior to MedSigLIP and its conversational counterpart, MedGemma-1.5. **Conclusions:** The findings highlight a trade-off between specialization and flexibility, where domain-specific models provide higher accuracy and stability, while general-purpose multimodal models offer greater accessibility, adaptability, and interactive reasoning, serving as complementary tools for retinal disease screening and clinical decision support.

## 1. Introduction

Diabetic retinopathy (DR) remains one of the leading causes of preventable blindness worldwide [1,2,3], with image-based early detection playing a critical role in reducing vision loss by enabling timely intervention. Recent advances in artificial intelligence (AI), particularly deep learning, have significantly improved automated retinal image analysis [4,5,6,7]. Particularly, emerging foundation models in ophthalmology show promise in transforming retinal disease screening by enabling data-efficient learning, improved generalization, and broader clinical applicability [8]. For instance, RETFound [9], a self-supervised foundational model pretrained on retinal images, can effectively adapt to downstream retinal tasks. EyeCLIP [10] and RetiZero [11] incorporate image–text alignment to enhance semantic understanding and zero-/few-shot capability in ophthalmic tasks. While these models achieve strong performance, they typically require extensive domain-specific data and task-specific adaptation.

In parallel, general-purpose foundation models, including large multimodal models (LMMs), have gained attention in a wide range of medical imaging tasks [12,13]. Unlike domain-specialized retinal encoders, these models are trained on broad multimodal corpora and can be queried via natural-language prompting, without task-specific parameter updates. Recent studies have begun to quantify their strengths and limitations in ophthalmology [14,15,16,17,18]. For instance, GPT-4 was evaluated on 422 ophthalmic “Clinical Challenge” cases with interleaved clinical text and images, achieving 38.4% accuracy for open-ended diagnosis and 57.8% for next-step multiple-choice reasoning [14]. ChatGPT-4 Plus (April 2024 model) was evaluated across ophthalmology diagnostic tasks, showing strength in text-based tasks but limitations when images are involved [15]. A binary classification study using the Retinal Fundus Glaucoma Challenge dataset found that multimodal ChatGPT-4 detected glaucoma with high specificity but lower baseline sensitivity, which improved markedly after cropping (with a trade-off in specificity) [16]. Additional evaluations of ChatGPT on fundus images (a photograph of the interior surface of the eye, specifically the retina [19]) have quantified diagnostic behavior and common failure modes in image-related use in ophthalmology [20,21,22,23,24]. Notably, Ayhan et al. [23] showed that in-context learning could approach the performance of domain-specific retinal models on the Indian Diabetic Retinopathy Image Dataset (IDRiD) [25] with carefully designed prompts. Together, these studies suggest that general-purpose multimodal models can perform clinically relevant image-based tasks in ophthalmology.

Despite these advancements, several open questions remain. First, it is unclear how newer generations of general-purpose multimodal models perform under zero-shot and few-shot prompting—without extensive prompt engineering—relative to both fine-tuned and embedding-based domain-specific retinal models. Second, though linear probing shows promise as an effective adaptation strategy for leveraging embeddings extracted from LMMs, its comparative advantages over fine-tuning in low-data regimes warrant further investigation. In this work, we present a comprehensive comparison of general-purpose and domain-specific multimodal foundation models for diabetic retinopathy classification using IDRiD. By comparing their performance, we clarified the relative strengths and limitations of these approaches through the following observations: (i) general-purpose multimodal foundation models, evaluated using zero-shot prompting, achieved performance comparable to fine-tuned domain-specific foundation models; (ii) few-shot prompting was not universally beneficial, requiring further optimization for consistent performance gains; (iii) for domain-specific foundation models, linear probing can match or outperform fine-tuning.

This work makes three key contributions beyond recent studies, including Ayhan et al. [23]: (a) we benchmarked newer-generation LMMs (GPT-5.2, Gemini 3 Flash, MedGemma-1.5) that were not evaluated in prior work, using simple, plain prompts rather than carefully optimized prompting strategies; (b) we provided a unified head-to-head comparison of prompt-based LMMs against fine-tuned and linear-probed domain-specific foundation models (RETFound, EyeCLIP, MedSigLIP) on the same dataset; and (c) we demonstrate that recent general-purpose models exhibited reduced dependence on elaborate prompt engineering, as evidenced by competitive zero-shot performance.

## 2. Materials and Methods

### 2.1. Dataset Description

All experiments were conducted on the publicly available image dataset IDRiD [25], which contains 516 color fundus images with a resolution of 4288 × 2848 pixels, acquired under mydriasis using a Kowa VX-10α digital fundus camera with a 50-degree field of view. Each image is graded from 0 (Normal) to 4 (Severe) according to the International Clinical Diabetic Retinopathy Severity Scale.

### 2.2. Experimental Setup

**Figure 1:**
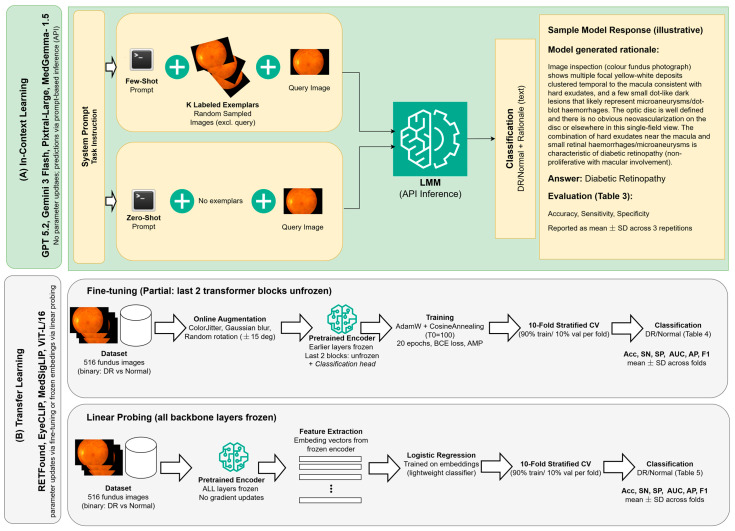
Overview of the experimental design. (**Upper panel**): general-purpose LMMs evaluated under zero-shot and few-shot prompting (In-Context Learning). (**Lower panel**): domain-specific vision and vision–language models evaluated through transfer learning, including fine-tuning and linear probing.

Following previous work [23], we formulated the task as a binary problem, where images labeled as grade 0 were considered normal (*n* = 168) or otherwise DR-positive (*n* = 348). For fine-tuning and embedding-based linear probing, model performance was evaluated using stratified 10-fold cross-validation, where in each fold approximately 90% of images (464–465 images) served as the training set and the remaining 10% (51–52 images) served as the validation/test fold; each image appeared in exactly one test fold across all 10 iterations. A separate held-out test set was not used because the small dataset size (516 images) would have further reduced the already limited training data and potentially compromised model convergence during fine-tuning. For prompt-based zero-shot experiments, each of the 516 images was evaluated as the query image; for few-shot experiments, exemplar sets were randomly sampled from the non-query images. We repeated the queries three times. Figure 1 shows an overview of the experimental design, including in-context learning (zero-shot and few-shot) for conversational LMMs (Gemini 3 Flash, GPT-5.2, Pixtral-Large-2411, and MedGemma-1.5 [26]) and transfer learning (fine-tuning and linear probing) for medicine/ophthalmology-specific models, which include EyeCLIP (https://github.com/Michi-3000/EyeCLIP) [10], RETFound (https://github.com/rmaphoh/RETFound) [9], and MedSigLIP v1.0.0 [26]. Table 1 provides an overview of all the models evaluated in this work and their descriptions.

### 2.3. Zero-Shot and Few-Shot Prompting

For general-purpose LMMs (Gemini 3 Flash, GPT-5.2, and Pixtral-Large), DR classification was performed using zero-shot and few-shot prompting strategies. This was also applied to MedGemma-1.5, a conversational medicine-specific chatbot that includes MedSigLIP as its image encoder. In the zero-shot setting, models were provided only with task instruction and the query fundus image, without any labeled example images. In the few-shot setting, five labeled fundus images were randomly selected from the remaining and were included in the prompt as exemplars to guide model inference. For each query image, few-shot exemplars were sampled from the remaining images (excluding the query image). No model parameters were updated in either setting, and all predictions were obtained through prompt-based inference alone (e.g., via API).

Specific parameters are set as follows: a temperature of 0.8 with a maximum output length of 2048 tokens for Gemini 3 Flash (“gemini-3-flash-preview”), default temperature with a maximum completion length of 4096 tokens for GPT-5.2 (“gpt-5.2-2025-12-11”), and a temperature of 0.7 and nucleus sampling with *top_p* = 0.9 for MedGemma-1.5-4B-IT [26]. Default decoding parameters were used for all other settings unless otherwise specified. For Pixtral-Large, the temperature was set to *t* = 0 (the recommended default for deterministic classification). For simplicity, we used the term general-purpose LMMs to refer to instruction-tuned, generative multimodal models that support prompt-based inquiry, even when they may incorporate domain-specific visual encoders (e.g., MedGemma-1.5).

### 2.4. Fine-Tuning of Domain-Specific Models

We evaluated the fine-tuning performance of multiple vision and vision–language foundation models, including RETFound_mae_natureCFP (ViT-L/16), MedSigLIP (ViT-So400m/14), EyeCLIP (ViT-L/14), and an ImageNet-pretrained ViT-L/16 baseline (see Table 1 for brief descriptions). Although EyeCLIP and MedSigLIP are designed for multimodal (image + text) inputs, we deliberately used their visual encoders as the focus of the study on image classification. In addition, this ensured a fair comparison to image-only models (RETFound, ViT-L/16). Fine-tuning was performed using a stratified 10-fold cross-validation protocol. To mitigate overfitting on the relatively small dataset, partial fine-tuning was adopted, wherein only the final two transformer blocks were trained while earlier layers remained frozen. Full fine-tuning was also explored but did not yield substantial performance improvements over partial fine-tuning and was therefore not emphasized in this study.

RETFound fine-tuning was performed using fundus images resized to 224 × 224 pixels using standard bicubic interpolation prior to training. The data augmentation details are described in Section 2.5. Validation images were normalized without augmentation. Models were optimized using AdamW with layer-wise learning-rate decay and a cosine annealing warm-restart scheduler (*T0* = 100). The learning rate was scaled as *base_lr* × (*batch_size* × *accum_steps*/256), where *accum_steps* denotes the number of gradient accumulation steps, a technique that increases the effective batch size without additional GPU memory; in our experiments, *accum_steps* = 2, yielding an effective batch size of 32. Training was performed for 20 epochs with a batch size of 16 using mixed-precision training. To address class imbalance (168 normal vs. 348 DR-positive), class-weighted binary cross-entropy loss was used, with *pos_weight* set to the ratio of negative to positive samples. Early stopping with a patience of 5 epochs was applied based on validation loss to mitigate overfitting. For partial fine-tuning, all backbone parameters were initially frozen, and only the classification head and the last two transformer blocks were unfrozen and updated during training.

The EyeCLIP ViT-L/14, MedSigLIP (google/medsiglip-448), and Vision Transformer (ViT-Large/16) vision encoder were fine-tuned using a linear classification head appended to the frozen backbone, with the final two transformer blocks unfrozen to allow task-specific adaptation. Training was conducted for 20 epochs using the AdamW optimizer with a learning rate of 1 × 10^−5^, 5 × 10^−5^, and 1 × 10^−4^, respectively, and the weight decay was set to 0.01. A Cosine Annealing with Warm Restarts scheduler (*T_0* = 100) was applied across training steps to modulate the learning rate. Binary cross-entropy with logits was used as the loss function given the binary classification objective. For MedSigLIP and ViT, mixed-precision training was enabled via PyTorch’s (v2.8.0) automatic mixed precision (AMP) with a gradient scaler to improve computational efficiency.

### 2.5. Data Augmentation

All fine-tuned models shared a common online data augmentation pipeline applied stochastically during training (i.e., augmented variants are generated on-the-fly at each epoch; the total number of unique images remains 516). The shared augmentation parameters were random ColorJitter (brightness, contrast, and saturation factors of 0.4; hue factor of 0.1), random Gaussian blur (*kernel size* = 3, applied with probability 0.5), and random rotation up to ±15 degrees. No geometric cropping or resizing augmentations were applied beyond what each pretrained model expects. Model-specific differences in preprocessing and normalization are summarized in Table 2.

### 2.6. Linear Probing

For linear probing experiments, pretrained image encoders were kept frozen, and feature embeddings were extracted from each model (namely, RETFound, MedSigLIP, EyeCLIP (ViT-L/14 or ViT-L/14@336px), and Vanilla ViT-L/16). A logistic regression classifier was then trained on these embeddings to perform binary classification. Performance was evaluated using the same stratified 10-fold cross-validation scheme as in the fine-tuning experiments.

### 2.7. Evaluation Metrics

Model performance was assessed using multiple standard classification metrics, including the accuracy, sensitivity, specificity, ROC-AUC, precision, and F1 score. For cross-validation experiments, results are reported as means ± standard deviations with 95% confidence intervals (CIs) assuming normal distributions. ROC-AUC and related threshold-based metrics were computed only for models that produced probabilistic outputs (e.g., not for prompt-based inquiries).

## 3. Results

### 3.1. Performance of General-Purpose Multimodal Models

Table 3 summarizes the performance of general-purpose LMMs evaluated using simple zero-shot and few-shot (five-shot in this case) prompting strategies. Results are reported across three independent repetitions, along with their averages and 95% CIs. In the zero-shot setting, Gemini 3 Flash achieved the highest average accuracy (88.5%) and MedGemma-1.5 came second at 88.2%, without meaningful improvement under the few-shot setting. GPT-5.2 achieved a zero-shot accuracy of 77.9% with very high specificity (99.6%), indicating strong performance in correctly identifying normal cases, while few-shot prompting improved the sensitivity by 6.3% at a minor cost to specificity. Pixtral-Large demonstrated lower zero-shot performance but benefited substantially from few-shot prompting, improving the average accuracy from 70.7% to 78.1%.

In addition to GPT-5 and Gemini models, we evaluated MedGemma-1.5-4B-IT, a medically oriented instruction-tuned LMM (Table 3). In the zero-shot setting, MedGemma-1.5-4B-IT achieved 88.2% accuracy (sensitivity 92.6%; specificity 79.2%). Few-shot learning slightly reduced sensitivity to 91.2% with comparable specificity (79.0%), resulting in an overall accuracy decrease of 1.0%.

### 3.2. Fine-Tuning Performance of Domain-Specific Models

The performance of domain-specific vision–language models following fine-tuning is reported in Table 4. Among the evaluated models, MedSigLIP achieved the strongest performance, with an accuracy of 94.8%, ROC-AUC of 98.1%, and F1 score of 96.0%. EyeCLIP also demonstrated robust performance, achieving an accuracy of 85.8%, ROC-AUC of 94.4%, and F1 score of 88.5%. In contrast, RETFound showed substantially lower accuracy, at 77.1%. A partially fine-tuned ViT-L/16 baseline achieved moderate performance, with an accuracy of 85.7%.

These results demonstrate that general-purpose and instruction-tuned multimodal models can achieve competitive performance in diabetic retinopathy classification with some ophthalmology-specific models using task-specific fine-tuning. However, their performance remained inferior to the MedSigLIP model designed for broad medical image analysis.

### 3.3. Linear Probing Performance

Table 5 presents the results of linear probing, where frozen image embeddings were extracted and classified using logistic regression under stratified 10-fold cross-validation. MedSigLIP again achieved the highest accuracy (94.4%) and F1 score (95.8%), comparable to its fine-tuned performance. Linear probing led to performance improvements for the two ophthalmology-specific models compared to fine-tuning: EyeCLIP by +2.3% (to 88.2%) and RETFound by +9.7% (to 86.8%).

A vanilla ViT-L/16 baseline achieved lower performance than domain-specific models but remained competitive, with an accuracy of 84.9%. These results indicate that linear probing effectively leverages pretrained representations and can outperform fine-tuning in certain cases, particularly for smaller datasets (IDRiD in our case).

### 3.4. Statistical Comparisons

Statistical significance tests were conducted for pairwise model comparisons on accuracy. For fine-tuned models, MedSigLIP significantly outperformed all other models: EyeCLIP (*p* < 0.001), ViT-L/16 (*p* < 0.001), and RETFound (*p* < 0.001). RETFound showed significantly lower performance than EyeCLIP (*p* = 0.002) and ViT-L/16 (*p* = 0.003) (Supplementary Table S1). The difference between EyeCLIP and ViT-L/16 was insignificant. For linear probed models, MedSigLIP again significantly outperformed any other models *(p* < 0.001), with no other model comparisons reaching significance (Supplementary Table S2).

Comparing adaptation strategies, linear probing significantly improved RETFound accuracy over fine-tuning (*p* < 0.001) (Supplementary Table S3). However, the improvements for other models remained insignificant, indicating that linear probing primarily benefits models with weaker fine-tuning performance.

Pairwise model comparisons among zero-shot models revealed significance (*p* < 0.001) between all comparisons, except for Gemini 3 Flash versus MedGemma-1.5 (the top 2 models) (Supplementary Table S4). The two models remained top in the 5-shot setting and still showed no significant difference (Supplementary Table S5). Between-shot comparisons showed that the transition from zero-shot to 5-shot prompting produced significant improvements for GPT-5.2 (*p* = 0.002) and Pixtral-Large (*p* = 0.003), but not for Gemini 3 Flash or MedGemma-1.5 (Supplementary Table S6).

### 3.5. External Validation on DeepDRiD

To assess model generalizability, we evaluated the models by testing their predictions on the DeepDRiD dataset [27]. For prompt-based models, we randomly sampled the images to match the size of IDRiD—168 Normal and 348 DR—to save API cost for financial consideration. For fine-tuning models and ensemble-based learning, we utilized all the 1600 labeled regular fundus images from DeepDRiD.

Among the prompt-based models (Table 6), MedGemma-1.5-4B-IT achieved the highest zero-shot accuracy (81.2%), substantially outperforming Gemini 3 Flash (73.8%), GPT-5.2 (61.0%), and Pixtral-Large (54.8%). Notably, few-shot prompting degraded MedGemma-1.5’s performance (−5.4%), while Gemini 3 Flash showed a small improvement (+1.6%). GPT-5.2 and Pixtral-Large both benefited from few-shot prompting, consistent with their IDRiD behavior.

Fine-tuned and linear-probed models (trained on the whole IDRiD set) were further tested on the whole DeepDRiD. For fine-tuned models (Table 7), MedSigLIP again achieved the highest accuracy (80.2%), followed by EyeCLIP (72.8%). RETFound exhibited extreme class bias with 98.4% sensitivity but only 0.8% specificity. Among linear-probed models (Table 8), MedSigLIP maintained its lead with 83.7% accuracy, while EyeCLIP dropped substantially to 55.4% with very low sensitivity (21.1%).

Overall, MedGemma-1.5 and MedSigLIP demonstrated the most robust cross-dataset generalization. The substantial performance drops observed for the other models underscore the importance of external validation and suggest that strong performance on a single dataset does not guarantee generalizability.

## 4. Discussion

In this study, we conducted a systematic comparison between general-purpose LMMs and domain-specific ophthalmic foundation models for DR classification on the IDRiD dataset and validated key findings on the external DeepDRiD dataset. Our results demonstrated that recent general-purpose models can achieve competitive performance in zero-shot and few-shot settings, in some cases matching or approaching fine-tuned domain-specific vision–language models (e.g., EyeCLIP), though remaining below the best-performing medical-specific model (namely MedSigLIP). However, domain-specific models continue to exhibit more consistent and higher peak performance, particularly when combined with representation-based adaptation strategies such as linear probing.

### 4.1. Comparison with Prior In-Context Learning Approaches

Our findings are closely aligned with recent work by Ayhan et al. [23], who showed that in-context learning with multimodal foundation models can approach the performance of specialized retinal models on the same IDRiD dataset. Their study demonstrated that carefully designed prompts and exemplar selection strategies enable general-purpose models to perform competitively without parameter updates. In contrast, our results indicate that new versions of the tested LMMs can achieve comparable performance using plain prompts, including simple zero-shot. This suggests that architectural and training advances in recent general-purpose models may reduce the dependence on elaborate prompt engineering for medical image analysis.

At the same time, our results reinforce the conclusion that performance consistency remains a challenge for prompt-based approaches. While zero-shot and few-shot prompting yielded strong average accuracy, sensitivity–specificity trade-offs varied across models, and improvements from few-shot prompting were not uniform. These observations highlight an inherent variability in prompt-driven inference that may limit reliability in clinical settings.

### 4.2. Comparison with RETFound: Vision-Only Domain-Specific Foundation Models

RETFound represents a strong vision-only, domain-specific foundation model trained via large-scale self-supervised learning on retinal images [9]. Consistent with prior reports, RETFound demonstrated solid performance when adapted to DR classification through fine-tuning [9]. In our experiments, linear probing substantially improved RETFound’s performance compared to fine-tuning, underscoring the effectiveness of frozen representation learning for small medical datasets.

Despite these strengths, RETFound and similar vision-only models require explicit training or adaptation to each downstream task. Their interpretability is largely derived from post hoc techniques such as saliency maps and relevance propagation [28,29], which, while informative, provide limited interaction and contextual reasoning. In contrast, general-purpose multimodal models offer flexible inference without task-specific retraining and enable interactive reasoning through natural language, albeit with lower peak accuracy and greater variability.

### 4.3. Comparison with EyeCLIP: Domain-Specific Vision–Language Models

EyeCLIP extends the foundation model paradigm by explicitly aligning ophthalmic images with clinical language through large-scale multimodal pretraining [10]. As demonstrated in prior work, EyeCLIP achieves strong zero-shot, few-shot, and supervised performance across multiple ophthalmic datasets and exhibits improved semantic interpretability through image–text alignment. In our study, EyeCLIP achieved robust performance when fine-tuned and further benefited from linear probing, reinforcing the value of multimodal representation learning in ophthalmology.

However, the strengths of EyeCLIP come at the cost of substantial domain-specific data collection and training complexity. In contrast, our results suggest that general-purpose multimodal models—trained without ophthalmology-specific objectives—can nevertheless achieve competitive performance on DR classification with minimal few-shot adaptation. This highlights a trade-off between specialization and flexibility: domain-specific multimodal models offer superior accuracy and consistency, while general-purpose models provide rapid deployment, broad applicability, and interactive reasoning capabilities.

### 4.4. Explainability and Clinical Implications

Explainability is a critical consideration for the clinical adoption of AI systems. Three complementary forms of interpretability are available across the model categories evaluated in this study. First, vision-only models such as RETFound rely primarily on visual attribution methods (e.g., Grad-CAM [28], saliency maps, and layer-wise relevance propagation [29]) to highlight salient retinal regions. Second, domain-specific vision–language models such as EyeCLIP provide semantically grounded explanations via image–text alignment, enabling textual descriptions of visual features that the model considers diagnostically relevant. Third, general-purpose multimodal foundation models enable clinicians to query model predictions using natural language and receive contextualized responses. While our work focused on classification performance, future work should extend to explainability evaluation, for example, through the recruitment of clinical experts to assess the extent to which the explanations align with clinical reasoning.

### 4.5. Limitations and Future Work

This study has some limitations. First, experiments were conducted on two datasets (IDRiD and DeepDRiD) with a binary DR classification formulation, which may limit generalizability. The binary classification scheme followed previous work by Ayhan et al. [23] with clinical relevance to screening workflows (referral vs. non-referral). However, given the significance of multi-grade DR severity classification in treatment planning, future work will extend the evaluation to multi-grade classification. Second, evaluation focused primarily on classification performance and did not include open-end diagnostic performance. Third, prompt-based evaluation is subject to inherent response variability due to stochastic decoding. To account for variability, we repeated each image query three times, reporting means with standard deviations and 95% CIs. In addition, we disclosed model versions and settings as well as prompts to ensure reproductivity. Finally, while our current evaluation focused primarily on image input, incorporating clinical text metadata alongside images via the multimodal pathways such as EyeCLIP and MedSigLIP represents a promising future direction.

## 5. Conclusions

In this study, we systematically compared general-purpose multimodal foundation models and domain-specific retinal foundation models for diabetic retinopathy classification on the IDRiD dataset and validated key findings on the external DeepDRiD dataset. Our results show that recent general-purpose LMMs can achieve competitive performance using plain zero-shot and few-shot prompting, in some cases approaching or matching the accuracy of fine-tuned domain-specific vision–language models. However, domain-specific models demonstrated more consistent performance and higher peak accuracy, particularly when adapted using linear probing in small-data settings. External validation on DeepDRiD revealed that MedSigLIP and MedGemma-1.5 maintained the strongest generalization, whereas other models exhibited notable performance degradation, underscoring the importance of multi-dataset evaluation. These findings highlight a trade-off between specialization and flexibility. While domain-specific foundation models remain preferable for applications requiring maximal accuracy and stability, general-purpose LMMs offer advantages in terms of ease of access, adaptability, and interactive reasoning. Together, general-purpose models may serve as valuable complementary tools to domain-specific models for retinal disease screening and clinical decision support.

## Figures and Tables

**Table 1:** Brief description of evaluated models, their modalities, and knowledge cutoff points for conversational variants.

Model Name(s)	Model Description	Modality	Knowledge Cutoff
GPT-5.2-2025-12-11	The GPT-5.2 generation provides state-of-the-art reasoning and multimodal processing for text and image inputs.	Conversational, Image + Text	31 August 2025
Gemini-3-Flash-Preview	Gemini 3 Flash preview model optimized for fast inference and deployment.	Conversational, Image + Text	January 2025
Pixtral-Large-2411	Large multimodal model integrating vision and language understanding.	Conversational, Image + Text	Not available
MedGemma-1.5-4B-IT	Google’s medical multimodal model specialized for clinical reasoning and instruction tuning.	Conversational, Image + Text	Not available
MedSigLIP v1.0.0 (ViT-So400m/14)	Google’s vision model designed for broad medical image analysis.	Image + Text	Not available
EyeCLIP (ViT-L/14, ViT-L/14@336px)	Model trained specifically on ophthalmic (fundus) imaging tasks.	Image + Text	Not available
RETFound_mae_natureCFP (ViT-L/16)	ViT-based model pretrained on retinal imaging datasets.	Image-only	Not available
ViT-L/16	Standard Vision Transformer baseline without domain specialization.	Image-only	Not available

**Table 2:** Model-specific preprocessing and normalization parameters.

Model	Input Resolution	Normalization	Preprocessing
RETFound	224 × 224	ImageNet (mean = [0.485, 0.456, 0.406]; std = [0.229, 0.224, 0.225])	Bicubic resize to 224 × 224
EyeCLIP	224 × 224	CLIP-specific (mean = [0.4815, 0.4578, 0.4082]; std = [0.2686, 0.2613, 0.2758])	Native CLIP preprocessing
MedSigLIP	448 × 448	Native model processor	Resizing disabled; native processor
ViT-L/16	224 × 224	Native model processor	Resizing disabled; native processor

Online augmentation (ColorJitter, Gaussian blur, random rotation) was identical across all models.

**Table 3:** Performance of general-purpose multimodal models.

Model	Accuracy (%)	Sensitivity (%)	Specificity (%)
GPT-5.2 (0-shot)	77.9 ± 0.4 [77.2, 78.6]	67.4 ± 0.6 [66.3, 68.5]	99.6 ± 0.3 [99.0, 100]
GPT-5.2 (5-shot)	81.5 ± 0.1 [81.3, 81.7]	73.7 ± 0.3 [73.0, 74.3]	97.8 ± 0.3 [97.2, 98.4]
Gemini 3 Flash (0-shot)	88.5 ± 0.0 [88.5, 88.5]	84.8 ± 0.0 [84.8, 84.8]	96.4 ± 0.0 [96.4, 96.4]
Gemini 3 Flash (5-shot)	85.1 ± 0.4 [84.4, 85.8]	80.0 ± 0.4 [79.2, 80.8]	95.6 ± 0.3 [95.0, 96.2]
Pixtral-Large (0-shot)	70.7 ± 0.7 [69.5, 72.0]	58.0 ± 1.0 [56.1, 59.9]	97.0 ± 0.0 [97.0, 97.0]
Pixtral-Large (5-shot)	78.1 ± 1.0 [76.3, 79.9]	74.2 ± 0.9 [72.6, 75.8]	86.1 ± 1.2 [83.8, 88.4]
MedGemma-1.5-4B-IT (0-shot)	88.2 ± 0.1 [88.0, 88.4]	92.6 ± 0.2 [92.3, 92.9]	79.2 ± 0.0 [79.2, 79.2]
MedGemma-1.5-4B-IT (5-shot)	87.2 ± 1.1 [85.2, 89.2]	91.2 ± 0.6 [90.1, 92.3]	79.0 ± 2.4 [74.5, 83.4]

Results reported as mean ± SD [95% CI] across 3 repetitions.

**Table 4:** Fine-tuning performance of domain-specific models.

Model	Accuracy (%)	Sensitivity (%)	Specificity (%)	ROC-AUC	Avg. Precision	F1 Score
RETFound	77.1 ± 6.2 [72.7, 81.6]	75.3 ± 7.2 [70.1, 80.5]	80.9 ± 8.0 [75.2, 86.6]	86.9 ± 5.9 [82.7, 91.1]	92.9 ± 4.4 [89.7, 96.0]	75.7 ± 6.4 [71.1, 80.3]
MedSigLIP	94.8 ± 2.4 [93.0, 96.5]	93.1 ± 2.7 [91.1, 95.0]	98.2 ± 2.9 [96.1, 100]	98.1 ± 2.2 [96.6, 99.7]	99.2 ± 0.8 [98.7, 99.8]	96.0 ± 1.9 [94.6, 97.4]
EyeCLIP	85.8 ± 3.9 [83.1, 88.6]	81.3 ± 5.2 [77.6, 85.1]	95.2 ± 3.8 [92.5, 97.9]	94.4 ± 2.5 [92.6, 96.2]	97.7 ± 1.0 [97.0, 98.3]	88.5 ± 3.4 [86.1, 90.9]
ViT-L/16	85.7 ± 4.6 [82.3, 89.0]	82.2 ± 5.5 [78.3, 86.1]	92.9 ± 5.4 [89.0, 96.7]	94.2 ± 2.8 [92.2, 96.2]	97.5 ± 1.2 [96.6, 98.3]	88.5 ± 4.1 [85.6, 91.4]

Results reported as mean ± SD [95% CI] across 10 folds.

**Table 5:** Linear probing performance of domain-specific models.

Model	Accuracy (%)	Sensitivity (%)	Specificity (%)	ROC-AUC	Avg. Precision	F1 Score
RETFound	86.8 ± 2.1 [85.3, 88.4]	91.9 ± 3.0 [89.8, 94.1]	76.2 ± 6.7 [71.4, 81.0]	93.3 ± 2.7 [91.3, 95.3]	96.7 ± 1.6 [95.6, 97.8]	90.4 ± 1.5 [89.3, 91.5]
MedSigLIP	94.4 ± 3.2 [92.1, 96.7]	95.1 ± 3.3 [92.7, 97.5]	92.8 ± 5.6 [88.8, 96.8]	97.9 ± 2.0 [96.4, 99.4]	99.1 ± 0.8 [98.6, 99.7]	95.8 ± 2.4 [94.0, 97.5]
EyeCLIP	88.2 ± 3.8 [85.5, 90.9]	91.4 ± 2.7 [89.4, 93.3]	81.6 ± 9.7 [74.7, 88.5]	95.6 ± 1.9 [94.2, 97.0]	98.2 ± 0.7 [97.6, 98.7]	91.3 ± 2.6 [89.4, 93.2]
ViT-L/16	84.9 ± 4.2 [81.9, 87.9]	89.4 ± 4.4 [86.2, 92.6]	75.6 ± 7.5 [70.3, 81.0]	92.5 ± 4.8 [89.1, 95.9]	96.6 ± 2.3 [94.9, 98.2]	88.9 ± 3.2 [86.6, 91.1]

Results reported as mean ± SD [95% CI] across 10 folds.

**Table 6:** Performance of general-purpose multimodal models on sampled DeepDRiD.

Model	Accuracy (%)	Sensitivity (%)	Specificity (%)
GPT-5.2 (0-shot)	61.0 ± 0.7 [59.8, 62.3]	45.6 ± 0.6 [44.5, 46.7]	93.1 ± 0.9 [91.4, 94.7]
GPT-5.2 (5-shot)	67.6 ± 0.7 [66.4, 68.9]	55.4 ± 0.7 [54.1, 56.6]	93.1 ± 0.9 [91.4, 94.7]
Gemini 3 Flash (0-shot)	73.8 ± 0.5 [72.9, 74.6]	73.2 ± 0.5 [72.2, 74.2]	74.9 ± 1.1 [72.9, 76.9]
Gemini 3 Flash (5-shot)	75.4 ± 0.8 [74.0, 76.9]	73.9 ± 0.6 [72.8, 75.0]	78.6 ± 3.0 [73.1, 84.1]
Pixtral-Large (0-shot)	54.8 ± 0.4 [54.0, 55.5]	43.3 ± 0.2 [43.0, 43.6]	78.6 ± 1.0 [76.7, 80.5]
Pixtral-Large (5-shot)	63.3 ± 0.8 [61.9, 64.7]	60.7 ± 1.1 [58.7, 62.7]	68.7 ± 0.3 [68.0, 69.3]
MedGemma-1.5-4B-IT (0-shot)	81.2 ± 0.0 [81.2, 81.2]	80.5 ± 0.0 [80.5, 80.5]	82.7 ± 0.0 [82.7, 82.7]
MedGemma-1.5-4B-IT (5-shot)	74.8 ± 0.7 [73.5, 76.1]	66.7 ± 1.3 [64.4, 69.0]	91.7 ± 1.6 [88.8, 94.6]

Results reported as mean ± SD [95% CI] across 3 repetitions.

**Table 7:** Performance of fine-tuned models based on IDRiD but tested on DeepDRiD.

Model	Accuracy (%)	Sensitivity (%)	Specificity (%)	ROC-AUC	AP	F1 Score
RETFound	54.9	98.4	0.8	59.5	64.6	36.2
MedSigLIP	80.2	81.6	78.4	88.2	91.0	82.0
EyeCLIP	72.8	65.0	82.5	82.2	86.0	72.6
ViT-L/16	65.5	66.1	64.7	72.4	79.8	68.0

**Table 8:** Performance of linear probed models based on IDRiD but tested on DeepDRiD.

Model	Accuracy (%)	Sensitivity (%)	Specificity (%)	ROC-AUC	AP	F1 Score
RETFound	65.0	42.4	93.0	79.9	83.6	57.3
MedSigLIP	83.7	81.9	85.9	91.2	93.1	84.8
EyeCLIP	55.4	21.1	97.9	77.1	80.9	34.4
ViT-L/16	54.4	57.3	50.8	57.3	62.9	58.2

## Data Availability

Python codes for API access to different LMM models (with prompts), model fine-tuning, and embedding-based linear probing have been deposited to GitHub: https://github.com/iqbalnaved/dr-classification-study, accessed on 13 May 2026.

## Supplementary Materials

The following supporting information can be downloaded at https://www.mdpi.com/article/10.3390/diagnostics16101504/s1, Table S1: Pairwise comparisons among fine-tuned models; Table S2: Pairwise comparisons among linear-probed models; Table S3: Fine-tuning vs. linear probing for each model; Table S4: Pairwise comparisons among zero-shot models; Table S5: Pairwise comparisons among 5-shot models; Table S6: Between shot comparisons.
